# An Extract of Pomegranate Fruit and Galangal Rhizome Increases the Numbers of Motile Sperm: A Prospective, Randomised, Controlled, Double-Blinded Trial

**DOI:** 10.1371/journal.pone.0108532

**Published:** 2014-10-02

**Authors:** Maja D. K. Fedder, Henrik B. Jakobsen, Ina Giversen, Lars P. Christensen, Erik T. Parner, Jens Fedder

**Affiliations:** 1 Laboratory of Reproductive Biology, Scientific Unit, Regional Hospital of Horsens, Horsens, Denmark; 2 Nerthus ApS, Lejre, Denmark; 3 Department of Chemical Engineering, Biotechnology and Environmental Technology, Faculty of Engineering, University of Southern Denmark, Odense, Denmark; 4 Department of Public Health, Section for Biostatistics, University of Aarhus, Aarhus, Denmark; 5 Centre of Andrology, Fertility Clinic, Department D, Odense University Hospital, Odense, Denmark; University of Kansas Medical Center, United States of America

## Abstract

Pomegranate fruit (*Punica granatum*) and galangal (*Alpinia galanga*) have separately been shown to stimulate spermatogenesis and to increase sperm counts and motility in rodents. Within traditional medicine, pomegranate fruit has long been used to increase fertility, however studies on the effect on spermatogenesis in humans have never been published. With this study we investigated whether oral intake of tablets containing standardised amounts of extract of pomegranate fruit and powder of greater galangal rhizome (Punalpin) would increase the total number of motile spermatozoa. The study was designed as a prospective, randomized, controlled, double-blinded trial. Enrolment was based on the mean total number of motile spermatozoa of two ejaculates. The participants delivered an ejaculate after 4–8 days of tablet intake and two ejaculates just before they stopped taking the tablets. Seventy adult men with a semen quality not meeting the standards for commercial application at Nordic Cryobank, but without azoospermia, were included in the study. Participants were randomized to take tablets containing extract of pomegranate fruit (standardised with respect to punicalagin A+B, punicalin and ellagic acid) and freeze-dried rhizome of greater galangal (standardised with respect to 1′S-1′-acetoxychavicol acetate) or placebo on a daily basis for three months. Sixty-six participants completed the intervention (active treatment: n = 34; placebo: n = 32). After the intervention the total number of motile spermatozoa was increased in participants treated with plant extracts compared with the placebo group (p = 0.026). After three months of active treatment, the average total number of motile sperm increased by 62% (from 23.4 to 37.8 millions), while for the placebo group, the number of motile sperm increased by 20%. Sperm morphology was not affected by the treatment. Our findings may help subfertile men to gain an improved amount of motile ejaculated sperm by taking tablets containing preparations of pomegranate fruit extract and rhizome of greater galangal.

**Trial Registration:**

ClinicalTrials.gov NCT01357044

## Introduction

In the Western world, a decline in sperm quality during the last 40 years has been suggested [1], [2], although the decrease may have been stabilized during the last decade [3]. Only 23% of Danish men have optimal semen quality and 15% are at high risk of needing fertility treatment in order to biologically father a child [3]. It is evident that any improvement in poor quality semen brings about an increased chance for obtaining pregnancy, also with regard to assisted reproductive technology. Intracytoplasmic sperm injection is often the only alternative offered to couples, where the male suffers from poor semen quality. This makes randomised controlled trials of supplements, drugs or diet advices, aimed to improve semen quality, important in order to create other solutions helpful for males with poor semen quality.

Pomegranate (*Punica granatum*) and greater galangal (*Alpinia galanga*) belong to a limited group of well-known edible plants, which show promising effects *in vivo* on rodent sperm quality without causing undesirable side effects [4]–[7]. The pomegranate fruit consists of a leathery pericarp containing numerous seeds, each covered by a juicy, translucent aril. Both pericarp and aril juice contains hydrolysable tannins, with the pericarp being the far most concentrated source [8]. In the pericarp, the ellagitannins punicalagin A and B predominates, while punicalins, which are partial hydrolysis products of punicalagins [9], constitute a minor fraction. The ellagitannins are only found in trace amounts in the aril juice [8]. Instead, other hydrolysable tannins prevail in the juice together with another important group of polyphenols, the anthocyanins [10]. Ellagitannins and, to a lesser degree, anthocyanins possess pronounced antioxidative activity *in vitro* [8]. Juice from *P. granatum* fruit has been shown to stimulate spermatogenesis, to increase sperm motility, and to decrease the percentage of abnormal sperm in rats after seven weeks of gavage [4]. An increase in sperm motility and concentration together with a decrease in abnormal sperm rate was observed after 6 weeks of oral administration of pomegranate juice extract to rats [5]. Further, oral administration of pomegranate pericarp extract has been shown effective in reversing induced spermatogenic disruption in rats [6]. The ginger-like rhizome of *A. galanga* contains a wide variety of semivolatile and volatile compounds, the dominating substances being a group of phenylpropanoids with 1′S-1′-acetoxychavicol acetate (ACA) as the most abundant. The phenylpropanoids have shown potent antioxidative activity *in vitro* [11], while rhizome extracts of *A. galanga* have shown anti-inflammatory effect *in vivo* [12]. Additionally, extracts of the rhizome of *A. galanga* have been show to increase sperm counts and motility in mice [7] and to increase serum testosterone levels in rats [13].

In this study it was investigated whether oral consumption of tablets with a standardised content of *P. granatum* extract and *A. galanga* powder would increase the total number of motile spermatozoa (TMSC) and sperm morphology, defined by strict criteria, in adult men with reduced semen quality. Since this is the first published clinical study investigating the effects of *P. granatum* and *A. galanga* on human semen quality, we chose to focus on the most important (number and motility) [14] and basic (morphology) semen parameters [15].

## Materials and Methods

The protocol for this trial and supporting CONSORT checklist are available as supporting information; see Checklist S1 and Protocol S1.

### Study design

The hypothesis was formulated prior to the study, and the power was estimated with the assumptions that the difference between the study and placebo group would be 5 million TMSC and SD = 2.3 million TMSC. If a significance level of 5% and a statistical power of 80% were given, at least 18 participants should be included in each group. However, due to the high level of uncertainty of the assumptions in this power estimation, nearly twice as many participants were enrolled.

The study was a prospective, randomised, placebo-controlled, double-blinded trial. The participants were randomised 1∶1 to daily treatment with tablets containing extract of pomegranate fruit (standardised with respect to punicalagin A+B, punicalin and ellagic acid) and freeze-dried rhizome of greater galangal (standardised with respect to 1′S-1′-acetoxychavicol acetate (ACA)) or placebo tablets, for 90 days. In addition to the two ejaculates delivered prior to intervention (baseline  =  mean of the two ejaculates), the participants delivered an ejaculate after 4–8 days of tablet intake and two ejaculates at the end of the study. Upon termination of the study, the participants completed a questionnaire in order to determine the occurrence of any negative or positive side effects.

### Study participants and semen collection

Participants were recruited over a one-year period by advertising in Nordic Cryobank (sperm bank) and local newspapers. Inclusion criteria were: Healthy adult men ≥18 years of age with a semen quality not meeting the standards for commercial application at Nordic Cryobank (i.e.>200 mio. TMSC in raw semen). Potential sperm donors, who fulfilled the requirements, completed a questionnaire about former illness and medicine consumption to ensure that the reason for inferior semen quality was not due to obvious medical reasons, such as cryptorchidism or genital tract infection.

Enrolment was based on two ejaculates delivered by masturbation within a time span of 7–14 days and with 3–7 days of abstinence before each sample delivery. The ejaculates were kept at or above room temperature (max 30°C) and analysed within one hour from ejaculation. Participants with azoospermia were excluded from the study. Seventy participants were enrolled, but four chose to withdraw due to logistic difficulties.

### Interventions

The daily active treatment consisted of 4 tablets with extract of *P. granatum* and 4 tablets with *A. galanga* (Punalpin, Nerthus ApS, Lejre, Denmark), two of each kind taken in the morning, and two of each kind in the evening. The placebo tablets were produced in two variations to visually match the two kinds of active treatment. Four of each kind of placebo tablets were taken daily, two of each kind in the morning and two of each kind in the evening. The participants of both groups received all the tablets at the beginning of the study in two separate containers. The daily active dose provided 1000 mg *P. granatum* extract and 764 mg *A. galanga* preparation, corresponding to 500–1000 mL high quality pomegranate fruit juice (based on the amount of characteristic ellagitannins) and approximately 1 g fresh greater galangal rhizome (based on the amount of ACA). For *P. granatum* this corresponded to: 106 mg punicalagin A (27 mg/tablet), 278 mg punicalagin B (69 mg/tablet), 4,7 mg punicalin (1,2 mg/tablet) and 9,6 mg ellagic acid (2,4 mg/tablet). For A. *galanga*, this corresponded to: 16 mg ACA (3.9 mg/tablet).

Initial screening of a range of commercially available extracts of *P. granatum* and *A. galanga* for their content of characteristic bioactive compounds showed that most extracts of *P. granatum* had either (i) very low levels of punicalagin A and B, punicalin and ellagic acid or (ii) low levels of punicalagin A and B and punicalin, but high levels of ellagic acid, indicating that the former compounds had been degraded to ellagic acid. This is in accordance with the study of Zhang *et al.* (2009) [16] who screened 27 commercial extracts of *P. granatum* and concluded that only 5 reflected the typical tannin profile of *P. granatum* while five extracts had no detectable tannins or ellagic acid. Our screening showed that the ethanolic fruit (pomace) extract P40P (Polinat, Las Palmas, Spain) had a fingerprint profile of punicalagin A and B, punicalin and ellagic acid, which was very similar to that of high quality pomegranate juice (data not shown) and was therefore selected for the present trial. P40P has been standardised to contain at least 35% punicalagin A+B and at least 40% punicosides (the total amount of punicalagins, punicalin, ellagic acid glycosides and ellagic acid).

Our screening of a number of commercial extracts of *A. galanga* revealed that the content of ACA was either very low or not detectable (data not shown). Therefore, we developed the following method for preparing an extract of *A. galanga* with high levels of ACA combined with very low levels of microbial counts: Fresh rhizomes of *A. galanga* imported from Thailand were purchased (Europa Frugt ApS, Valby, Denmark). The rhizomes were stored at approximately 5°C during transport. Upon arrival the stems were removed and the rhizomes were split longitudinally and transferred to a −24°C freezing chamber. After a few days of freezing the *A. galanga* rhizomes were freeze-dried. The water content in the rhizomes were 3–5% following drying. The rhizomes were transferred to gas tight sealed bags (VMPET12/Adhesive/LLDPE100, total 114 µm, WJ Packaging solutions, Canada) in nitrogen atmosphere (0–3% O_2_) for 14 days. Subsequently the foil bags with rhizomes were placed in a heating oven for 3 hours at 78±2°C in order to further decrease the number of microorganisms. Prior to incorporation in tablets, the dry rhizomes were grated on a standard Co-mill pulveriser.

Tablets were produced containing either 191 mg of the above-mentioned dried powder of *A. galanga* or 250 mg of *P. granatum* extract. The *P. granatum* tablets additionally contained sodium bicarbonate, silicon dioxide, microcrystalline cellulose, syloid AL1, magnesium stearate and, for coating, propylene glycol, titanium dioxide, talc, and pharmacoat 615.

The *A. galanga* tablets contained talc, microcrystalline cellulose, syloid AL1, magnesium stearate, plus coating as just described added shellac and copper complexes of chlorophylls for colouring purpose. The placebo tablets contained 461 mg microcrystalline cellulose, syloid AL 1 and magnesium stearate plus coating, and the placebo tablets matching the *A. galanga* tablets also added copper complexes of chlorophylls.

The levels of marker compounds in the tablets were measured 1, 5, 12 and 21 months after production in order to monitor any degradation.

### Outcome

The primary outcome was change in semen quality during the 3 month long treatment period, from baseline to follow-up, expressed as the total (progressive and non-progressive) motile sperm count (TMSC). The TMSC was defined as: Ejaculate volume × spermatozoa concentration × percentage of motile spermatozoa [15].

The secondary outcome was change in number of morphologically normal sperm based on WHO's strict sperm morphology criteria [15]. The outcomes were calculated as the difference between the follow-up value and the baseline value.

### Chemical analysis of tablets

The level of ACA in the tablets was determined by gas chromatography-flame ionization detection (GC-FID) and gas chromatography-mass spectrometry (GC-MS), while levels of punicalagin A and B, punicalin, and ellagic acid were determined by analytical high-performance liquid chromatography with diode-array detection (HPLC-DAD).

For GC sample preparation, 15 tablets were ground in a mortar and 5.00 g of the ground tablets were mixed in 50 mL 99.9% ethanol in a 100 mL flask with screw cap. The mixture was subjected to a sonication bath for 20 min. Approximately 1 mL of this solution was filtered through a HPLC filter (Whatman Puradisc 13 syringe filters (0.2 µm), Sigma-Aldrich, Germany) into GC-vials before GC-FID and GC-MS analysis, respectively. GC-FID analysis was performed on an Agilent 6890 GC series (Agilent Technologies, Hørsholm, Denmark) and a Thermo Scientific DSQ II Single Quadrupole GC/MS system (Thermo Scientific, CA, USA) was used for GC-MS analysis. Separations were performed on a ZB-Wax plus column (60 m×0.25 mm internal diameter (i.d.), df = 0.25 µm liquid phase, Phenomenex Denmark, Værløse, Denmark) using the following oven temperature program: 80°C for 1 min, from 80°C to 220°C at 5°C/min, followed by a constant temperature at 220°C for 20 min. Helium was applied as carrier gas with a flow rate of 1.2 mL. The injection volume was 1 µL with a split ratio of 1∶50 (injector temperature 200°C). ACA was identified by GC-MS (70 eV) based by comparison of retention time (R_t_) and mass spectrum with an authentic standard of ACA (PhytoLab GmbH & Co. KG, Vestenbergsgreuth, Germany) and quantified by GC-FID (FID operating at 300°C) based on a calibration curve of ACA covering the concentration range occurring in the samples. All analyses were performed in duplicates with a relative standard deviation of less than 1%.

For HPLC sample preparation, 15 tablets were ground in a mortar and 30 mg of the ground tablets were mixed with 20 mL distilled water in a 50 mL flask with screw cap. The mixture was subjected to a sonication bath for 20 min. The dissolved tablet sample was filtered through a HPLC filter (0.2 µm) before HPLC analysis. Analytical HPLC-DAD analysis was carried out on an Agilent 1200 Series analytical HPLC system (Hørsholm, Denmark) equipped with a DAD operating from 190 to 600 nm. Polyphenols were monitored at 258 nm, and UV spectra were recorded between 190 and 600 nm. Separations were performed on a Luna 3 µm C18(2) 100 Å, LC end-capped column (150 mm×4.6 mm internal diameter, 3 µm particle size, Phenomenex Denmark, Værløse, Denmark). The mobile phases consisted of 0.2% phosphoric acid in water as solvent A and 100% acetonitrile as solvent B, and the polyphenols were separated using the following solvent gradient: 0 min (5% B), 7 min (30% B), 8 min (70% B), 13 min (70% B), 15 min (5% B), and 17 min (5% B). Solvents were degassed before use. The flow rate was 1.0 mL/min, the column temperature was 40°C, and the injection volume was 10 µL. Stock standard solutions of punicalagin A and B (ChromaDex, Boulder, CO, USA), punicalin (Stanford Chemicals, Irvine, CA, USA), and ellagic acid (Sigma-Aldrich, Munich, Germany), respectively, were prepared in water:methanol (80∶20, v:v) at a concentration of 1 mg/mL. Water was used for further dilution of the stock standard solutions. The polyphenol standards were used for identification (R_t_ on HPLC and UV spectra) and quantification (calibration curves covering the concentration range occurring in the samples). All analyses were performed in duplicates with a relative standard deviation of less than 5%.

### Analysis of sperm motility

Number of motile sperm in each semen sample was determined using a Makler counting chamber (Sefi Medical Instruments Ltd., Haifa, Israel) by two laboratory technicians both blinded to the study. Each semen sample was allowed to liquefy and was analysed within one hour from delivery. Conditions during analysis were standardised to room temperature (22°C) and a preheated Leica microscope. The sperm in a minimum of 10 grid squares were counted, and when present, a minimum of 200 sperm per sample were counted in total. Intra-observer coefficient of variation was assessed in intervals and never found to exceed the accepted difference between independent replica counts [15]. In the cases of replica counts, the first reading was included in the study.

### Analysis of sperm morphology

Number of morphologically normal sperm was determined by blinded strict morphology analysis of ethanol-fixated Papanicolaou stained semen smears. The staining procedure was performed as recommended by the WHO 5^th^ edition [15]. On each smear, if present, 400 sperm were counted and rated either abnormal or normal based on WHO's strict sperm morphology criteria [15]. All the morphology analyses were performed by the same person and within one month in order to minimize rating variations.

### Randomisation

Randomisation of the participants was performed by a computer-based block-randomisation sequence formed with allocation 1∶1 and block size 10, meaning that for each 10 participants enrolled an equal number of participants are randomized to each group. Tablets with active compounds and placebo had identical appearances. The tablets were stored in separate plastic containers with consecutive numeration matching the participant randomisation number. Participants meeting the inclusion criteria received the tablets upon returning a written consent. The randomisation code was kept unknown to anyone except the one person, who managed the randomisation, and not revealed until the study was finished and all primary statistical calculations were performed.

### Statistical analyses

The primary analysis was a comparison of the mean increase in TMSC between the treatment group and placebo group. The mean increase in TMSC was compared using an unequal variance t-test, taking into account possible variance heterogeneity between the two groups and deviation from normal distribution [17]. As a confirming analysis, all 95% confidence intervals and p-values were calculated by the bootstrap method [18]. In a secondary analysis adjustments were made in a linear regression model for age and BMI, both dichotomized by the median value. In a sensitivity analysis the adjustment for age and BMI were also performed using restricted cubic spline with each 2 knots in the linear regression model. For all comparative analyses, p<0.05 was considered statistically significant.

### Approvals

The study was approved by the Danish Data Protection Agency (J.nr. 2012-41-5441) and the Scientific Ethics Committee of Middle Jutland, Denmark (M-20100247) and registered in ClinicalTrials.gov (ID NCT01357044). All participants provided informed written consent.

## Results

### Study participants

**Figure 1:**
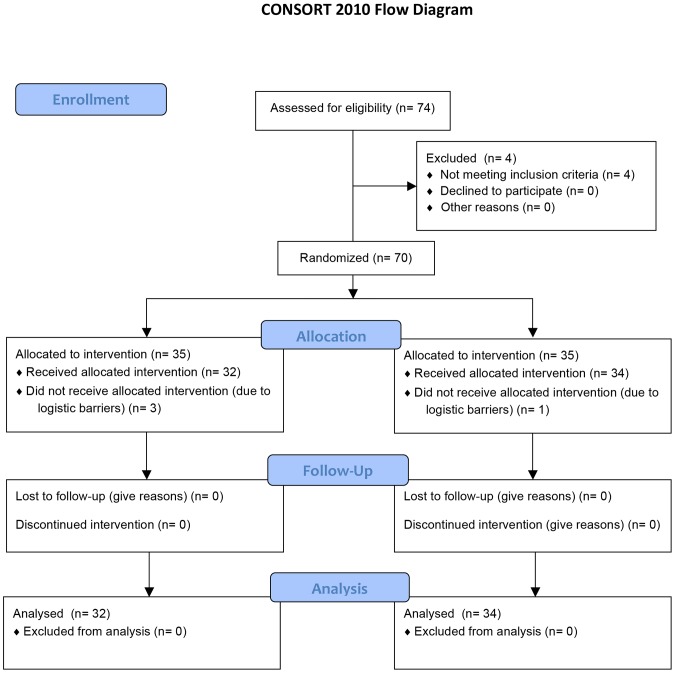
Flow diagram. Consort diagram showing the flow of the study participants.

From May 2011 to September 2012, 70 adult men (mean age: 29 years) were included and randomised, and 66 received active treatment (n = 32) or placebo (n = 34), Figure 1. The mean age for the group receiving active treatment was 30±7 years, and for the group receiving placebo treatment the mean age was 28±6 years. Body mass index (BMI) and life style factors did not differ significantly between the two groups (Table 1). In each group one participant had only one testicle (the other one presumably removed due to cryptorchidism), and two participants in each group reported previous infection with chlamydia. Fifty-three of the participants delivered a baseline semen sample with at least one abnormal semen parameter (volume, concentration, motility) according to the new standards from 2010 (≥1.5 mL and ≥15 million/mL and ≥40% motile spermatozoa) [15]. One study participant in the treatment group had only one follow-up value.

**Table 1:** Demographics and life style factors.

	Active treatment	Placebo
	(n = 32)	(n = 34)
Age (years)	30.6±7.3 (CI: 28.1–33.2)	28.1±6.1 (CI: 26.1–30.2)
BMI	25.8±3.8 (CI: 24.5–27.1)	25.8±4.7 (CI: 24.2–27.4)
Smoking, n	5 (15.6%)	5 (14.7%)
Coffee drinking (≥4 cups/day), n	9 (28.1%)	3 (8.8%)
Alcohol drinking (≥14 units/week), n	2 (6.3%)	2 (5.9%)
Sauna regularly (monthly), n	2 (6.3%)	1 (2.9%)

Demographic data and life style factors of the participants showing a similar pattern for the two study groups. Participants were asked to state basic life style (smoking, coffee drinking, alcohol drinking and how often they attended sauna). Age at the time of baseline ejaculate delivery was calculated from social security numbers, and BMI was calculated from given weights and heights.

### Total motile sperm count (TMSC)

**Figure 2:**
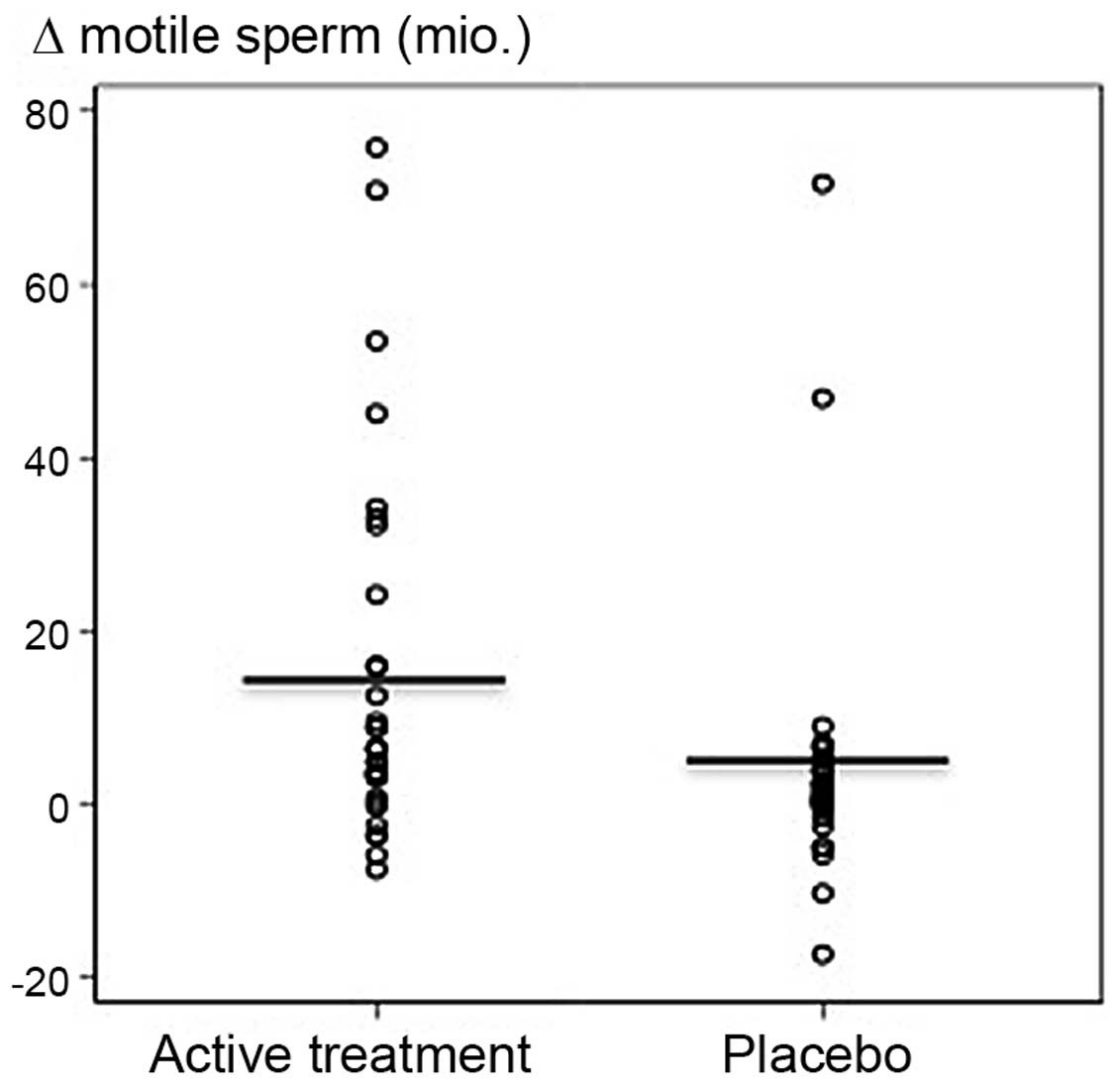
Differences in total motile sperm counts (TMSCs). Plot of the differences in TMSCs from baseline to follow-up for each participant in the active treatment receiving the combination of *P. granatum* fruit extract and *A. galanga* rhizome powder and the placebo group. Follow-up – baseline (Y-axis) represents the calculated differences in TMSC between the follow-up TMSCs following 90 days of administration of either the dry preparation of *A. galanga* and the *P. granatum* extract or the placebo and the corresponding TMSCs prior to administration (baseline). Horizontal bars indicate mean.

Treatment with standardised amounts of *P. granatum* extract and *A. galanga* powder for three months induced a 62% increase in the average total number of motile sperm (from 23.4 millions to 37.8 millions) which was a significantly higher increase than in the placebo group (20%: from 19.9 millions to 23.9 millions), p = 0.026 (Table 2). Bootstrap analysis showed very similar results (p = 0.021). Figure 2 shows the individual plots of the differences between follow-up and baseline TMSC. There was no increase in the number of motile sperm after one week of tablet intake for either of the groups (Table 2). When data were adjusted for age and BMI (using division at the median), the difference in increase in TMSC remained statistically significant (Table 2). Similar results were obtained when adjusting for age or BMI using restricted cubic spline (data not shown).

**Table 2:** Total motile sperm counts (TMSCs).

	Treatment		Crude		Adjusted	
	Active	Placebo				
	(n = 32)	(n = 34)	Difference	P-value*	Difference	P-value*
	Mean ±SD (95% CI)	Mean ±SD (95% CI)				
Baseline	23.4±25.1 (14.3; 32.4)	19.9±22.7 (12.0; 27.8)	3.5 (−8.3; 15.3)	0.56	4.4 (−8.2; 17.0)	0.49
Initiating treatment	23.6±25.4 (14.4; 32.8)	20.1±22.9 (12.1; 28.1)	3.5 (−8.4; 15.4)	0.56	4.7 (−8.0; 17.3)	0.46
Follow-up	37.8±39.5 (23.6; 52.1)	23.9±27.8 (14.2; 33.6)	14.0 (−3.0; 30.9)	0.10	14.2 (−3.8; 32.2)	0.12
Initiating treatment - baseline	0.2±6.0 (−1.9; 2.4)	0.2±9.9 (−3.3; 3.6)	0.1 (−4.0; 4.1)	0.98	0.3 (−3.9; 4.5)	0.89
Follow-up - baseline	14.5±21.3 (6.8; 22.1)	4.0±15.2 (−1.3; 9.3)	10.5 (1.3; 1 9.7)	0.026	9.8 (0.2; 19.5)	0.047

TMSCs for the treatment groups receiving the combination of *P. granatum* fruit extract and *A. galanga* rhizome powder or placebo. The results are shown unadjusted and adjusted for age and BMI.

*The difference in TMSC between the two groups was analysed by an unequal variance t-test. In the adjusted analyses the participants were divided in two groups at the median according to age and BMI.

Although defined as normal semen quality, it has been shown that time to pregnancy increases, when the sperm concentration does not exceed 40×10^6^/mL [19]. Subanalyses of our dataset show a clear trend of larger increase in TMSC in the active treatment group compared to the placebo group when looking at participants with very low baseline TMSC. The statistical significance is maintained when looking at the subgroup of participants with baseline TMSC at or below 40 million (active treatment: n = 27, placebo: n = 28) (p = 0.02), however due to the number of participants in the study, the statistical power is lost when baseline TMSC is further lowered.

To be mentioned as secondary results, the power was not sufficient to reveal a possible effect on motility (per cent motile sperm) alone (p = 0.2), but the concentration of motile spermatozoa also increased significantly in the active treatment group compared with placebo (p = 0.030; 3.8 mio./mL in active treatment vs. 1.1 mio./mL in placebo).

Two of the participants in the treatment group were experiencing influenza-like symptoms during the study period. For both these participants, TMSC did not improve.

### Sperm morphology

Strict sperm morphology assessment did not reveal statistically significant difference between the treatment and the placebo groups (Table 3). Very similar results were obtained using an unequal variance t-test (Table 3) and the bootstrap method (data not shown).

**Table 3:** Sperm morphology.

	Treatment			
	Active	Placebo		
	(n = 32)	(n = 34)	Difference	P-value*
	%±SD (95% CI)	%±SD (95% CI)		
Baseline	5.2±3.2 (4.0; 6.3)	4.9±3.0 (3.8; 5.9)	0.3 (−1.3; 1.9)	0.70
Follow-up	5.4±3.4 (4.2; 6.7)	5.0±2.8 (4.0; 5.9)	0.5 (−1.1; 2.0)	0.55
Follow-up – baseline	0.3±1.8 (−0.4; 0.9)	0.1±1.8 (−0.6; 0.7)	0.2 (−0.7; 1.1)	0.71

Morphologically normal sperm for the treatment groups receiving the combination of *P. granatum* fruit extract and *A. galanga* rhizome powder or placebo.

*The difference in number of morphologically normal sperm between the two groups was analysed by an unequal variance t-test.

### Compliance and safety

Microbiological activity in both the *P. granatum* extract and the *A. galanga* powder were below the EU standards for herbal medicine, even prior to the applied heating procedure of *A. galanga*, which further decreased the microbiologic counts (data not shown).

Counting of the remaining tablets in all bottles at the end of the study showed that more than 85% of the participants had taken more than 80% of the tablets. Thirty-eight (57.6%) of the participants returned a questionnaire regarding side effects and state of health during the study period. Possibly unrelated to the treatment, three of the responders (two in the treatment group and one in the placebo group) reported influenza-like symptoms. One participant from the active treatment group reported side effects in the form of irregular defecation, diarrhoea, and increased flatulence, but nevertheless wished to complete the treatment.

### Level of marker compounds

**Figure 3:**
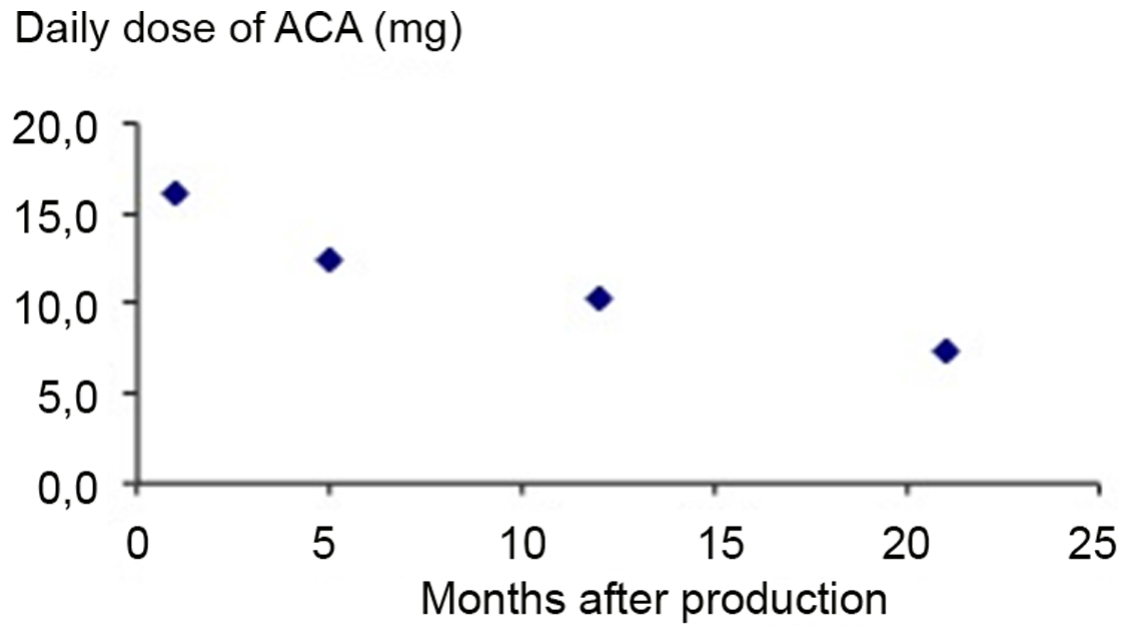
ACA content in tablets. Amount of 1′S-1′-acetoxychavicol acetate (ACA) in the *A. galanga* tablets within 21 months of production date.

The amount of ACA in the tablets was reduced during the period in which monitoring took place. The initial level of ACA was reduced by approx. 50% 21 months after tablet production (Figure 3). The levels of punicalagin A and B, punincalin and ellagic acid in pomegranate tablets were unchanged during the same period (data not shown). Since the recruitment, and thus the starting points for the participants, was forthcoming, the first recruited participants were taking tablets with a higher content of ACA in the *A. galanga* composition. For the first 34 participants included, the average increase in normal sperm counts was 74% (p = 0.045) (data not shown), possibly, but not certainly, reflecting the higher amount of ACA.

## Discussion

This study revealed that the combination of *P. granatum* fruit extract and *A. galanga* rhizome powder (Punalpin) significantly increased the total number of motile sperm in men with a semen quality, which is reduced or low within the normal range, after three months of daily treatment.

Maturing sperm move from the testis to the epididymis, in which they spend in average six days prior to release during ejaculation [20]. Since no difference in the number of motile sperm was seen after the first week of intake of standardised extract of *P. granatum* and *A. galanga* powder, we propose that the combination exerts its positive effect during spermatogenesis rather than on the epididymal environment. This is in concordance with the rodent study of Türk *et al*., who concluded that pomegranate juice stimulates spermatogenesis, increases sperm motility and decreases the number of abnormal sperm in rats in a dose-dependent manner [4]. However, the mechanism behind the observed improvement in semen quality still has to be clarified. One factor that may contribute considerably to the observed effects is the high levels of antioxidants present in both plants. A few studies have demonstrated a significant reduction in enzymatic and non-enzymatic antioxidant activity in seminal plasma of infertile men compared with fertile men and a positive influence of intake of antioxidants on the semen quality in humans [21], [22]. Additionally, the consumption of pomegranate juice or fruit has resulted in significant rises in plasma antioxidant capacity [23], [24]. Lastly, although not proved as causative factors, a few non-intervention studies have demonstrated statistical association between intake of fruits and greens and decreased risk of reduced semen quality [25], [26].

### Strengths and limitations

The present study was solidly designed. However, the relatively wide range in baseline values among the participants constitutes a weakness of the study. With a more homogenous study population, the 95% confidence intervals of the changes in TMSC would be expected to narrow down, followed by stronger statistical results.

It is also a weakness of the study that pregnancy rate was not an outcome parameter, but the participants were not recruited on the basis of a wish for pregnancy.

Lastly, information on reactive oxygen species level and antioxidant capacity would have been highly relevant.

### Comparison with other studies

Since this is, to our knowledge, the first study to address the effect of *P. granatum* and *A. galanga* on sperm quality in men, the magnitude of effect can only be compared with clinical human studies of other supplements. These studies mainly assess the effects of antioxidants.

The polyphenols in pomegranate exhibit significant antioxidant activity *in vitro* [10]. Mansour *et al*. administrated extract of *P. granatum* to rats and suggested that the observed decrease in the level of malondialdehyde, a by-product of lipid peroxidation, could be related to the accompanied decrease in abnormal sperm rate and increase in sperm motility, i.e. an antioxidant effect protecting the sperm cell membrane from lipid peroxidation [5]. Although numerous studies have addressed the question of whether oral intake of antioxidants is indeed beneficial for semen quality [22], several authors of recent studies and reviews conclude that more large well-designed studies are needed in this area. However, despite lack of consensus between studies and lack of evidence for improvement in the most important outcome parameter, pregnancy rate, the majority of studies find a positive effect of various antioxidants on semen quality, mainly measured by sperm concentration and motility [27]–[29]. Recently, an oral supplement of various antioxidants was shown to decrease the number of immotile sperm and to significantly increase the TMSC in men undergoing fertility treatment [30]. In another study, daily intake of sesame was shown to increase sperm count and motility [31]. In both these studies, the interval of semen analysis from baseline to follow-up was at least two months, leaving no information on whether the supplements had an effect on epididymal sperm. Nevertheless, both studies referred to the antioxidant effect as the major reason for sperm quality improvement. In the study on the effects of sesame on sperm quality, sperm morphology was also assessed, but in accordance with our present findings there was no improvement in sperm morphology after three months of taking the sesame supplement [31].

Several studies have shown that oxidative stress in the seminal fluid, i.e., excess levels of reactive oxygen species (ROS) compared with the antioxidant levels, causes decreased sperm quality via two mechanisms: 1) Some ROS act as free radicals and cause damage to the sperm cell membrane thereby decreasing sperm motility and 2) the free radicals may cause damage to the sperm DNA [22]. Both *P. granatum* and *A. galanga* contain significant levels of antioxidants that may fully or partly explain the effect observed in this study on sperm quality. The antioxidant activity of *P. granatum* might be due to the abundant polyphenols present in the fruit as their chemical structure indicates that they can act as direct and indirect antioxidants. The bioavailability of polyphenols is, however, of major concern when talking about the direct antioxidant effect of these compounds because the concentration in the systemic system is often too low to have any significant direct antioxidant effect *in vivo*. Therefore, the preventive effects of polyphenols against, e.g., oxidative stress are probably related to their ability to activate the endogenous antioxidant defence system; hence acting as indirect antioxidants. Polyphenols containing *ortho*-phenol groups as those found in pomegranate fruit can be metabolised by phase 2 enzymes to electrophilic *ortho*-quinones, which is a prerequisite for activating the endogenous antioxidant defence system [32], [33]. The antioxidant properties of the most abundant polyphenols in *P. granatum* are therefore expected to be due to their indirect antioxidant activity.

Hence, the fact that the TMSC did not increase after approximately one week of tablet intake in the present study does not prove against an antioxidant effect of the combination of *P. granatum* fruit extract and *A. galanga* rhizome powder. It rather suggests a possible effect of indirect antioxidants. In addition, *P. granatum* polyphenols may also exert modulatory effects in cells through selective actions on different components of the intracellular signalling cascades such as hormones and regulatory proteins that are vital for cellular functions such as growth and proliferation [34], [35]. This may also explain the observed effects of the *P. granatum* fruit extract and *A. galanga* rhizome powder combination.

As for the polyphenols in *P. granatum*, the major bioactive constituent of *A. galanga*, the 1′S-1′-acetoxychavicol acetate (ACA), also acts as an indirect antioxidant, although the chemical structure is unrelated to the *P. granatum* polyphenols. ACA is also a strong electrophile [36], which seems to explain many of the reported bioactivities of this natural product. ACA has been shown to enhance phase 2 enzyme activity and to regulate the glutathione metabolism by activating the endogenous antioxidant defence system [37]–[40] essential for its antitumor and chemopreventive effects [36], [41]–[46].

### Conclusion and future studies

This study indicates that intake of tablets containing preparations of extract of *P. granatum* fruit pomace and the rhizome of *A. galanga* (Punalpin) may help men in gaining an improved amount of motile ejaculated sperm.

Running chemical analyses of the tablets confirmed the presence of several potentially bioactive compounds, e.g. antioxidants, however at present we do not know, which of these compounds may be responsible for the increase in TMSC. Furthermore, it has yet to be investigated whether the combined effect of *P. granatum* and *A. galanga* is synergistic or additive. The mechanism by which the combination of *P. granatum* and *A. galanga* causes increase in the number of motile sperm should be investigated in future studies, where also pregnancy rate and time to pregnancy should be outcome measures. Such a study will expectedly justify the recommendation of the commercial product in clinical practice.

## Supporting Information

Checklist S1 **Supporting CONSORT checklist.**
(DOC)

Protocol S1 **Original detailed protocol for the study approved by the local Ethics Committee.**
(DOC)

Background Data S1 **The raw background data.**
(XLS)
