# Supplementary material for: An Extract of Pomegranate Fruit and Galangal Rhizome Increases the Numbers of Motile Sperm: A Prospective, Randomised, Controlled, Double-Blinded Trial
Source: PLoS One. 2014 Oct 2;9(10):e108532. doi: 10.1371/journal.pone.0108532 (PMC4190413; doi:10.1371/journal.pone.0108532)
Supplement: Protocol S1 — Original detailed protocol for the study approved by the local Ethics Committee. (DOC) [file pone.0108532.s002.doc]

**Alternative treatment of reduced semen quality with plant extracts**

**Participants:**

**Laboratory of Reproductive Biology, Scientific Unit, Regional Hospital of Horsens**

Jens Fedder, associate professor, consultant, PhD

**Nerthus ApS**

Henrik Byrial, cand.agro., PhD

Ina Giversen, cand.hort., PhD

**Nordic Cryobank, Aarhus**

Peter Bower, cand.scient., PhD

**Institute for Biostatistik, Aarhus University**

Erik Parner, cand.scient., PhD

**Aim:**

The aim of the project is to investigate whether daily intake of two plant extracts for a period of three months can improve reduced semen quality in men.

The causes of reduced semen quality may be many. It is the aim of this project to find a treatment, which can be used in cases where reduced semen quality is environment and coupled to oxidative stress.

**Background:**

Reduced semen quality is a big and recognized problem in many western countries, not the least in Denmark, where it is estimated that up to 40% of young men is affected to some extend (1). To this day the focus has been on symptomatic treatment with assisted reproduction. This method is relatively expensive from a social perspective and often causes considerable inconvenience for the female part. Therefore, a treatment, which can improve the semen quality and decrease the need for assisted reproduction, is in high demand.

Plant ingredients against reduced semen quality

The project is based on a thorough and systematic search in the scientific literature with the aim to encircle plant extracts with a positive effect on the semen quality in human and/or animals. The criteria for choosing extracts for testing in a clinical trial was:

1. The plant extract should have shown a positive effect on semen quality in human and/or animals.
2. The plant extract should be approved as article of food in Denmark or the EU.
3. The plant extract must not have shown serious side effects in previous trials.

Based on these criteria two plant extracts was encircled:

Greater galangal (*Alpinia galanga* (Willd.) L.)

Pomegranate (*Punica granatum* L.)

Existing literature on these two plants was assessed with focus on effect on semen quality and other physiological properties, chemical substances, and safety.

*The effect of greater galangal on semen quality*

The effect of greater galangal on semen quality has been addressed in experiments with mice (2), where intake of the ethanolic extract for three months resulted in significantly improved motility of spermatozoa (P < 0.01) and an increased number of spermatozoa (P < 0.05). Additionally, an experiment with rats showed that intake of greater galangal increased the level of serum testosterone (3). A sufficiently high serum testosterone level is crucial for normal sperm production. The active component of greater galangal is not known, but it is possibly one or more phenylpropanoids, a group of components characteristic for greater galangal (4). The present studies do not indicate any safety risk with the intake of greater galangal (2, 5).

*The effect of pomegranate on semen quality*

The effect of pomegranate on semen quality has been addressed in experiments with rats (6), where intake of juice for seven weeks resulted in significantly increased sperm concentration (P < 0.01), improved motility (P < 0.05), and a decreased frequency of abnormal sperm (P < 0.05).

A different experiment on rats showed that a single component from pomegranate, ellagic acid, was able to counteract the negative effect on semen quality caused by cisplatin (7). Also in this study the oxidative balance in semen and serum was significantly improved.

In addition, juice from pomegranate has been shown to improve the oxidative balance in human serum (8, 9), which may be due to a high level of antioxidants, particularly ellagitannins and ellagic acid (10). One of the animal experiments indeed suggests that ellagitannins and ellagic acid play a central role in the improvement of the semen quality (7). The present studies do not indicate any safety risk with the intake of pomegranate (8, 11, 12), only risks of allergic reactions, which may appear following intake of many other fruits (13) cannot be excluded.

*In summary*

We have increased to ingredients, greater galangal and pomegranate, which both may improve semen quality, and which both are approved as food in the EU.

Causes of reduced semen quality

No unambiguous explanation exists to why reduced semen quality has become an increasing problem in Denmark and other western countries (14, 15). Among other factors, smoking during pregnancy (16), hormone disrupting components (17), obesity (18), and oxidative stress (19) have been linked to reduced semen quality.

In this project it is hypothesized that oxidative stress plays a substantial role in the development of reduced semen quality (20). Oxidative stress may appear due to environmental influences or health conditions and can be identified by a high level of reactive oxygen species (21). High levels of reactive oxygen species may damage the sperm membrane and DNA, and as a result the sperm will become defect (22). Oxidative stress may be neutralized by antioxidants, which inhibit the formation of reactive oxygen species (19).

Greater galangal and pomegranate both contain a high level of various antioxidants (23, 10). It is therefore reasonable to assume that these two ingredients have the ability to neutralize oxidative stress and as a result improve semen quality.

**Methods:**

**Design:** The study will be carried out as a randomized, double-blinded, placebo-controlled trial with two arms of equal size.

**Recruitment:** Participants will be reruited via the sperm bank Nordic Cryobank. Participants must be at least 18 years of age. Potential participants will be given written information and a declaration of consent at the sperm bank, and concomitantly they will be offered an elaborating conversation with Jens Fedder. If the man – after time to think it over – decides to participate, oral and written consent is obtained. On a regular basis approximately 1-2 men per week are declined as sperm donors in Nordic Cryobank due to reduced semen quality. Men who do not fulfil the donor criteria with respect to sperm count are offered a second semen analysis, and in the case the second sample remains at an equal standard the man is offered participation. Two analysed ejaculates prior to the treatment are required.

**The trial:**

When a potential participant has been given oral and written information about the trial and given his written consent, biostatistician professor Erik Parner will randomize him to get tablet containing either extracts of pomegranate and greater galangal or placebo tablets. The participant will concomitantly receive a questionnaire, which he is encouraged to fill out and return just prior to the cure. At the end of the three months of daily intake of tablets the participants will deliver two ejaculates with 4-10 days of interval. The treatment will be continued until the last ejaculate is delivered.

In addition to the start- and end-ejaculates, the participants are asked to deliver one ejaculate 4-8 days after treatment start to provide information on whether a possible effect can be measured after a short treatment time.

The ejaculates can be collected in Aarhus (where many of the potential participants expectedly live) but will be examined in the laboratory of Scientific Unit of Reproduction Biology, Horsens Regional Hospital. With the delivery of the last ejaculate the participant will receive kr. 1500 corresponding to kr. 300 per ejaculate. If a participant leaves the trial before the three months have passed, he will receive kr. 300 per delivered ejaculate. An experienced laboratory technician will together with Jens Fedder manage: the recruitment of participants, informing the participants about the trial, handling the hand-over of tablets, and administrating and coordinating the hand-in and analysis of ejaculates. The fact that the practical work is all handled by one experienced laboratory technician ensures that all analyses are carried out as similarly as possible.

The active treatment consists of a daily intake of 2x 1 gram of dried ethanolic extract of greater galangal and dried pomegranate corresponding to 500 mL of fresh pomegranate juice. The extracts are standardized from HPLC-measures of marker components. Both extracts are given as tablets. The placebo treatment holds daily intake of a corresponding number of tablets containing inactive components.

**Inclusion criteria:**

The only inclusion criteria are that the men should have a semen quality below semen donor level and be otherwise healthy.

**Exclusion criteria:**

Men with azoospermia, cryptorchidism, or symptoms of infection in genital tract cannot participate in the trial.

**Statistical analyses:**

Changes in the total number of spermatozoa will be compared between the two groups, which are receiving active treatment and placebo treatment, respectively, using relevant statistical analyses.

**Number of participants / power estimation:**

With a significance level of 5%, a statistical power of 80%, and a confidence interval of 2 million motile sperm, at least 18 participants should be included in each group (Erik Parner, Institute for Biostatistics, AU). However, we request permission to include 100 participants, thus 50 participants per arm.

**Semen analysis:**

The effect of the treatment is evaluated from measurements of the following classic semen parameters: concentration (sperm/mL), motility, and morphology. The measurements will be performed according to the WHO guidelines (24).

The total number of motile sperm (ejaculate volume x concentration of sperm x per cent of motile sperm) is a combined measure of semen quality. All ejaculates will be discarded immediately after analysis.

**Preparation and dosage of plant components:**

The participants are receiving greater galangal as a dried capsulated ethanolic extract, because the before mentioned animal experiments was based on such extract (2, 3). Pomegranate will be prepared as dried and capsulated juice.

The daily dose of galangal extract will be set at 2 g. This is in fact a lower dose than that used in the experiment with mice (2) (which would give a dose corresponding to 8 g for a man of 80 kg). The lower dose is chosen in order to minimize potential side effects such as gastric irritation, and because the effective dose of the related drogue ginger often is kept at this level (14).

The daily dose of dried pomegranate juice will be set at an amount corresponding to 500 mL of fresh juice. This amount is higher than that used in the before mentioned rat experiment (6) and is thus expected to be sufficient when thriving to reach a measurable effect.

We request that information about the identity, preparation, and dosage of the plant extracts will be kept confidential.

**Risks and precautions:**

Measurements of the chosen semen parameters (concentration, motility, and morphology) are routinely assessed at the location and require no further precautions.

**Etic:**

The participants will be informed about all aspects of the trial both orally and in writing. The extensive oral information will be given by Jens Fedder, and the informed consent will be collected after a couple of weeks to ensure the participant’s rights.

The project includes the inconvenience that participants have to meet and deliver fresh ejaculates several times.

The project may possibly be in use for the participants.

The project is in accordance with the declaration of Helsinki-II. The folder “Before you decide” from the local Scientific Ethics Committee will be given to the participants together with the project information for participants.

The project will be reported to the Danish Data Protection Agency.

**Economy:**

The project is financed with kr. 595,000 granted by the Centre for Science and Research of Alternative Treatment (VIFAB). Laboratory facilities are pre-existing and free to our disposal, and Aarhus University has granted 10 hours of statistical counselling.

**Publishing:**

The results of the trial will be published in a relevant international journal. Both positive and negative results will be published.

**References:**

1. Andersen, A.G., Jensen, T.K., Carlsen, E., Jorgensen, N., Andersson, A.M., Krarup, T., Keiding, N., and Skakkebak, N.E. (2000): High frequency of sub-optimal semen quality in an unselected population of young men. *Human Reproduction* 15, 2, 366-372.

2. Qureshi, S., Shah, A.H., and Ageel, A.M. (1992): Toxicity Studies on Alpinia-Galanga and Curcuma-Longa. *Planta Medica* 58, 2, 124-127.

3. Islam, M.W., Zakaria, M.N.M., Radhakrishnan, R., Liu, X.-M., Ismail, A., Chan, K., and Al-Attas, A. (2000): Galangal (*Alpinia galanga* Willed.) and Black seeds (*Nigella sativa* Linn.) and sexual stimulation in male mice. *Journal of Pharmacy and Pharmacology* 52 (Suppl.), 278-278.

4. Matsuda, H., Morikawa, T., Managi, H., and Yoshikawa, M. (2003): Antiallergic principles from Alpinia galanga: Structural requirements of phenylpropanoids for inhibition of degranulation and release of TNF-alpha and IL-4 in RBL-2H3 cells. *Bioorganic & Medicinal Chemistry Letters* 13, 19, 3197-3202.

5. Qureshi, S., Shah, A.H., Ahmed, M.M., Rafatullah, S., Bibi, F., and Al-Bekairi, A.M. (1994): Effect of Alpinia galanga treatment on cytological and biochemical changes induced by cyclophosphamide in mice. *International Journal of Pharmacognosy* 32, 2, 171-177.

6. Turk, G., Sonmez, M., Aydin, M., Yuce, A., Gur, S., Yuksel, M., Aksu, E.H., and Aksoy, H. (2008): Effects of pomegranate juice consumption on sperm quality, spermatogenic cell density, antioxidant activity and testosterone level in male rats. *Clinical Nutrition* 27, 2, 289-296.

7. Turk, G., Atessahin, A., Sonmez, M., Ceribasi, A.O., and Yuce, A. (2008): Improvement of cisplatin-induced injuries to sperm quality, the oxidant-antioxidant system, and the histologic structure of the rat testis by ellagic acid. *Fertility and Sterility* 89, 1474-1481.

8. Aviram, M., Rosenblat, M., Gaitini, D., Nitecki, S., Hoffman, A., Dornfeld, L., Volkova, N., Presser, D., Attias, J., Liker, H., and Hayek, T. (2004): Pomegranate juice consumption for 3 years by patients with carotid artery stenosis reduces common carotid intima-media thickness, blood pressure and LDL oxidation. *Clinical Nutrition* 23, 3, 423-433.

9. Rosenblat, M., Hayek, T., and Aviram, M. (2006): Anti-oxidative effects of pomegranate juice (PJ) consumption by diabetic patients on serum and on macrophages. *Atherosclerosis* 187, 2, 363-371.

10. Gil, M.I., Tomas-Barberan, F.A., Hess-Pierce, B., Holcroft, D.M., and Kader, A.A. (2000): Antioxidant activity of pomegranate juice and its relationship with phenolic composition and processing. *Journal of Agricultural and Food Chemistry* 48, 10, 4581-4589.

11. Forest, C.P., Padma-Nathan, H., and Liker, H.R. (2007): Efficacy and safety of pomegranate juice on improvement of erectile dysfunction in male patients with mild to moderate erectile dysfunction: a randomized, placebo-controlled, double-blind, crossover study. *International Journal of Impotence Research* 19, 6, 564-567.

12. Pantuck, A.J., Leppert, J.T., Zomorodian, N., Aronson, W., Hong, J., Barnard, R.J., Seeram, N., Liker, H., Wang, H.J., Elashoff, R., Heber, D., Aviram, M., Ignarro, L., and Belldegrun, A. (2006): Phase II study of pomegranate juice for men with rising prostate-specific antigen following surgery or radiation for prostate cancer. *Clinical Cancer Research* 12, 13, 4018-4026.

13. Eriksson, N.E., Werner, S., Foucard, T., Moller, C., Berg, T., Kiviloog, J., Norrlind, K., Soderberg, M., and Wihl, J.-A. (2003): Self-reported hypersensitivity to exotic fruit in birch pollen-allergic patients. *Allergology International* 52, 4, 199-206.

14. Carlsen, E., Giwercman, A., Keiding, N., and Skakkebaek, N.E. (1992): Evidence for Decreasing Quality of Semen During Past 50 Years. *British Medical Journal* 305, 6854, 609-613.

15. Swan, S.H., Elkin, E.P., and Fenster, L. (1997): Have sperm densities declined? A reanalysis of global trend data. *Environ.Health Perspect.* 105, 11, 1228-1232.

16. Jensen, T.K., Jorgensen, N., Punab, M., Haugen, T.B., Suominen, J., Zilaitiene, B., Horte, A., Andersen, A.G., Carlsen, E., Magnus, O., Matulevicius, V., Nermoen, I., Vierula, M., Keiding, N., Toppari, J., and Skakkebaek, N.E. (2004): Association of In Utero Exposure to Maternal Smoking with Reduced Semen Quality and Testis Size in Adulthood: A Cross-Sectional Study of 1,770 Young Men from the General Population in Five European Countries. *American Journal of Epidemiology* 159, 1, 49-58.

17. Daston, G.P., Gooch, J.W., Breslin, W.J., Shuey, D.L., Nikiforov, A.I., Fico, T.A., and Gorsuch, J.W. (1997): Environmental estrogens and reproductive health: A discussion of the human and environmental data. *Reproductive Toxicology* 11, 4, 465-481.

18. Hammoud, A.O., Gibson, M., Peterson, C.M., Meikle, A.W., and Carrell, D.T. (2008): Impact of male obesity on infertility: a critical review of the current literature. *Fertility and Sterility* 90, 4, 897-904.

19. Sikka, S.C. (1996): Oxidative stress and role of antioxidants in normal and abnormal sperm function. *Front Biosci.* 1, e78-e86.

20. Pasqualotto, F.F., Sharma, R.K., Nelson, D.R., Thomas, A.J., and Agarwal, A. (2000): Relationship between oxidative stress, semen characteristics, and clinical diagnosis in men undergoing infertility investigation. *Fertil.Steril.* 73, 3, 459-464.

21. Tremellen, K. (2008): Oxidative stress and male infertility - a clinical perspective. *Human Reproduction Update* 14, 3, 243-258.

22. Aitken, R.J., Gordon, E., Harkiss, D., Twigg, J.P., Milne, P., Jennings, Z., and Irvine, D.S. (1998): Relative impact of oxidative stress on the functional competence and genomic integrity of human spermatozoa. *Biol.Reprod.* 59, 5, 1037-1046.

23. Juntachote, T. and Berghofer, E. (2005): Antioxidative properties and stability of ethanolic extracts of Holy basil and Galangal. *Food Chemistry* 92, 2, 193-202.

24. WHO (2010): *WHO laboratory manual for the examination of human sperm-cervical mucus interaction.* Cambridge University Press, Cambridge, UK.
